# One-day voiding diary in the evaluation of Lower Urinary Tract Symptoms in children

**DOI:** 10.1590/S1677-5538.IBJU.2022.0375

**Published:** 2022-11-20

**Authors:** Hanny Helena Masson Franck, Ana Carolina S. Guedes, Yago Felyppe S. Alvim, Thamires M. S. de Andrade, Liliana Fajardo Oliveira, Lidyanne Ilidia da Silva, André Avarese de Figueiredo, José de Bessa, José Murillo B.

**Affiliations:** 1 Universidade Federal de Juiz de Fora Departamento de Cirurgia da Faculdade de Medicina Juiz de Fora MG Brasil Departamento de Cirurgia da Faculdade de Medicina – Universidade Federal de Juiz de Fora (UFJF), Juiz de Fora, MG, Brasil; 2 Universidade Federal de Juiz de Fora Escola de Enfermagem Juiz de Fora MG Brasil Escola de Enfermagem – Universidade Federal de Juiz de Fora (UFJF), Juiz de Fora, MG, Brasil; 3 Faculdade de Ciências Médicas e da Saúde de Juiz de Fora Escola de Fisioterapia Juiz de Fora MG Brasil Escola de Fisioterapia – Faculdade de Ciências Médicas e da Saúde de Juiz de Fora (HMTJ/SUPREMA), Juiz de Fora, MG, Brasil; 4 Universidade Estadual de Feira de Santana Departamento de Cirurgia da Faculdade de Medicina Feira de Santana BA Brasil Departamento de Cirurgia da Faculdade de Medicina – Universidade Estadual de Feira de Santana (UEFS), Feira de Santana, BA, Brasil

**Keywords:** Enuresis, Lower Urinary Tract Symptoms, Urinary Incontinence, Child

## Abstract

**Introduction:**

Voiding diary (VD) is an important tool in the evaluation of children with voiding symptoms. Voiding frequency, maximal voided volume (MVV), average voided volume (AVV) and nocturnal volume (NV) can be extracted and are valuable in diagnosing and monitoring these disorders. Recently, ICCS has reduced the period of data recording on VD from 3 to 2 days. We hypothesized that one day voiding diary would be enough for guiding treatment.

**Materials and Methods:**

Children with overactive bladder (OAB) and primary monosymptomatic enuresis (PMNE) were oriented to fulfill a 3-day VD. Data obtained from VD were evaluated for the first day (1dVD), the first two days (2dVD), and all 3 days (3dVD) and compared according to the MVV, AVV, frequency, NV and expected bladder capacity (EBC). The Friedman, Student's t test and the Fisher's exact was used. ANOVA was used for multiple comparisons. We also used Pearson correlation test.

**Results:**

Ninety-eight children were included, 59 had PMNE and 30 OAB. Frequency, AVV and VN were similar regardless how many days the voiding episodes were recorded. Only MVV was higher by a mean of only 32 mL on 3dVD compared to 1dVD. A 1dVD has a sensitivity of 93,9% and a positive likelihood ratio of 2.2. As for the correlation of MVV and EBC it was observed that in 83% of children, MVV was lower than EBC. MVV corresponds to 67% and 69% of EBC in children with PMNE and OAB, respectively.

**Conclusion:**

We believe that 1dVD is sufficient to assess these children. It has a high sensitivity and good correlation to 3dVD in evaluating these children. Bladder capacity in this population, evaluated by maximum voided volume, was close to 68% of that obtained by the EBC.

## INTRODUCTION

Lower urinary tract dysfunctions (LUTD) are disorders that can occur during the storage and voiding phases of micturition in the absence of neurological disease or lower urinary tract obstruction (1). Overactive bladder (OAB) is the most common type, being one of the most prevalent urinary disorders in childhood and defined as a condition that affects children presenting urinary urgency with or without incontinence and may or may not be associated with frequency (2).

Primary monosymptomatic nocturnal enuresis (PMNE) is characterized by involuntary voiding during sleep and is another prevalent disorder in childhood, affecting about 10 to 15% of children aged five to six years and is also included in the group of LUTD. OAB and PMNE are frequently associated with emotional and behavioral problems in children and directly affects their quality of life and many are still at high risk of suffering some type of punishment and can cause impacts on family members who live with the child (3–6). Therefore, clinical diagnosis and safe management of such disorders are of great importance, as well as instituting and monitoring the most appropriate treatment in order to minimize the short- and long-term consequences (7).

Voiding diary (VD) is an extremely useful tool that allows to draw a profile of the urinary routine, determining characteristics of bladder function in adults and children and is characterized by being a simple, non-invasive, low-cost method, free from complications, which best reproduces voiding habits (8, 9). Important measures such as voiding frequency, maximal voided volume (MVV), average voided volume (AVV) and nocturnal volume (NV) are easily extracted from VD and are valuable in diagnosing and monitoring these diseases (1, 2). According to the International Children Continence Society (ICCS), VD is one of the three diagnosis tests included in the so called non-invasive urodynamics, that also includes ultrasound with the evaluation of post-voided residual and uroflowmetry (1). The same study, ICCS has reduced the period of data recording on VD from 3 to 2 days. Our hypothesis is that one day voiding diary is enough for guiding treatment.

Studies in adult women have shown a strong correlation between the MVV obtained from a VD and the volume obtained when a strong voiding desire was referred during an urodynamics test, reinforcing the importance of the VD as a non-invasive alternative to urodynamics (10). Although VD is being reported in most of recent studies on LUTD, the analysis of the data obtained from it has shown great unconformities. Although VD is being reported in most of recent studies on LUTD, the analysis of the data obtained from it has shown great unconformities. Parents often complain of the difficulty of collecting all voiding episodes, drinking and bowel movements data for three, or even, two days as previously proposed (1, 11).

Therefore, the aim of the present study is to evaluate whether recording data in a VD for only one day would be enough to guide treatment, and also to evaluate and compare the maximum voided capacity to the expected bladder capacity (EBC) calculated by the formula proposed by Koff et al. (12).

## MATERIALS AND METHODS

A cross-sectional observational study was carried out in our LUTD clinic. The sample consisted of children aged 5 to 14 years presenting with OAB and PMNE who were instructed to fulfill a three-day VD as part of their assessment.

Parents and children were instructed on the objectives and methods of the study and after agreeing with them, they were invited to sign the informed consent and assent. The study was approved by the institution ethics committee under the number 1796620 and registered in the RBR- 3w2mxmw.

The data obtained from the VD were evaluated on the first day (1dVD), the first two days (2dVD) and all 3 days (3dVD) and compared according to the MVV, AVV, voiding frequency and NV, which includes night volume, measured by asking the parents to put a diaper in their children for the night and weighting it in the morning, added to the volume of the first morning void.

Children and adolescents with kidney, neurological and psychiatric diseases, secondary nocturnal enuresis, as well as conditions associated with LUTD such as diabetes or use of diuretic medications, and those whose parents did not agree to participate in the study, did not fill out correctly the VD, or did not perform any of the requested tests were not included in the study.

Before being included in the study, parents and children answered a structured questionnaire as well as the DVSS score (13), including daytime and nighttime questions on voiding function and habits, and were submitted to clinical evaluation. A kidney and bladder ultrasound were done to evaluate the urinary tract and post-voided residual and a uroflowmetry was done to evaluate voiding pattern. Those with high post-voided residual on ultrasound or an interrupted or staccato curve on uroflowmetry were not included in the study. The urotherapist nursing staff oriented on how to fulfill the 3 days VD and a dry night diary for 14 consecutive nights.

After the child returned with the VD and test results, those who fulfilled inclusion criteria were included in the study. Data from VD were analyzed by the urotherapist nurse. MVV was considered the highest voided volume excluding first morning void. Average voided volume was the average of all voiding, also excluding first morning voids. Voiding frequency was the mean frequency during the analyzed period and nocturnal volume was calculated adding the first morning void to the weight of the diaper. The formula adopted to calculating EBC used in this study was that proposed by Koff and suggested by ICCS (EBC= [age (yrs)+1] ×30 mL) (1, 12).

Quantitative data was expressed as mean ± standard deviation (SD) while qualitative variables were expressed as absolute values, percentages, or proportions. The Friedman or Student's t test were used to compare continuous variables, and the Fisher's exact or chi-square test was used for categorical comparisons. All tests were 2-sided with p <0.05 considered statistically significant. ANOVA was used for multiple comparisons. We also used Pearson correlation test to correlate 1dVD to 3dVD. Considering 3dVD as a reference test, sensitivity, specificity, overall accuracy, and predictive values, are described to 2dVD and 1dVD on estimates of low MVV. Analysis was performed using commercially available statistical software (GraphPad Prism, version 8.03 for Windows, San Diego California USA).

## RESULTS

A total of 98 children aged 8.23 ± 2.26 years (53% male) were included. Of these, 59 had PMNE with a mean age of 8.58 ± 2.35 years of age, being 30 boys (50.84%) and 39 presented OAB with a mean age of 7.72 ± 2.05 years old being 22 boys (56.41%).

**Figure 1:**
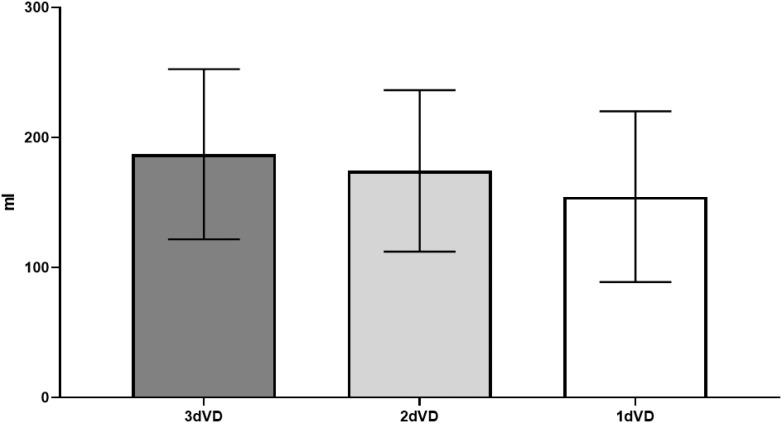
Comparison between Mean Maximum voided volume (MVV) obtained from 3 days (3dVD), 2 days (2dVD) and 1day (1dVD) voiding diary.

Frequency, AVV, and NV were similar, regardless of how many days VD was recorded (Table-1). The mean of difference for MVV was 19.8 mL higher in 2dVD compared to 1dVD (p<0.001) and 12.6 mL higher in 3dVD compared to 2dVD (p<0.001). Comparing 3dVD to 1dVD, it was 32.1 mL higher (p <0.01) (Table-1 and Figure-1). Pearson test showed a good correlation for MVV between 1dVD and 3dVD (r=0.82; CI: 0.74-0.87; p<0.001).

**Table 1:** Comparison of voiding frequency, MVV, AVV and NV in the 3dVD, 2dVD and 1dVD calculations of the VD.

Voiding Diary (number of days) vs. Voiding Parameters (Mean ? SD)				
	3dVD	2dVD	1dVD	p-value*
Frequency (voids/day)	6.6 ± 2.3	6.6 ± 2.2	6.8 ± 2.5	0.960
MVV (mL)	184.8 ± 65.9	172.2 ± 62.6	152.7 ± 65.3	0.0001
AVV (mL)	103.5 ± 39.2	104.5 ± 46.2	102.9 ± 46.9	0.133
NV (mL)	269.0 ± 148.0	290.5 ± 128.5	275.3 ± 121.9	0.356

* p value was < 0.001 when paring all groups for MVV (3dVD vs. 2dVD, 3dVD vs. 1dVD, and 2dVD vs. 1dVD)

MVV = Maximum Voided Volume; AVV = Average Voided Volume; NV = Nocturnal Volume; 3dVD = Three days Voiding Diary; 2dVD = Two days Voiding Diary; 1dVD = One day Voiding Diary; SD = Standard Deviation

**Figure 2:**
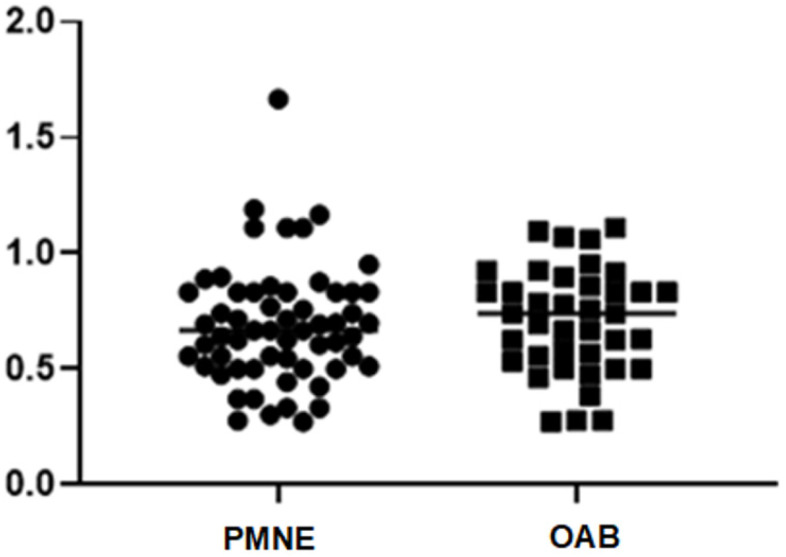
Ratio between Maximum Voided Volume (MVV) obtained for 3 days voiding diary and Expected Bladder Capacity (EBC). Observed was 67% of EBC for PMNE children and 69% of EBC for OAB children.

A discrepancy was observed between MVV and EBC in both groups. For the PMNE group the mean EBC was 287.29 ± 70.51 mL while the mean MVV was 191.02 ± 67.41 mL obtained on the 3dVD, 173.98 ± 63.99 mL for 2dVD, and 165.51 ± 67.20 mL for 1dVD. MVV corresponded to 67% of EBC when a 3-day VD was collected, 61% for 2dVD, and 58% for 1dVD. Similarly, in the OAB group, the mean EBC was 263.08 ± 61.74 mL and MVV was 181.41 ± 62.65 mL for 3dVD, 175.00 ± 60.01 mL for 2dVD, and 138.00 ± 60.34 mL for 1d VD, which corresponded to 69%, 67%, and 53% of EBC for one, two-, and three-days voiding diary, respectively. Considering only the 3dVD the maximum voided volume obtained was 67% of EBC for PMNE children and 69% of EBC for OAB children (Figure-2).

Considering 3dVD as the reference test to identify lower bladder capacity, especially that bellow 65% of EBC, (1) 1dVD has sensitivity of 93.87% (83.13 to 96.72%), specificity of 57.14% (42.21% to 71.18%), and an overall accuracy of 75.5% (65.8% to 83.6%, CI 95%). The positive and negative predictive values are 68.66% (61.14% to 75.31%) and 90.32% (75.23% to 96.63%), respectively.

If the data is collected for two consecutive days the sensitivity increases to 97.96% (89.15% to 99.95%), specificity to 87.75% (75.23% to 95.37%), and the overall accuracy to 92.9% (85.8% to 97.1%, CI 95%). The positive and negative predictive values are 88.89% (79.06% to 94.43%) and 97.73% (86.04% to 99.67%).

## DISCUSSION

Objective measurements of lower urinary tract symptoms are a clinical challenge. Although VD is routinely used in clinical practice for the primary assessment of children and adults, there are few studies on the use of VD in children.

The present study demonstrates that the number of days on which VD is performed, whether 1, 2 or 3 days, does not influence the analysis of voiding frequency, AVV and NV. In the other hand, MVV was lower when VD was collected for only one day, this difference represents 19.5 mL in comparison to 2dVD, which is the proposed number of days for VD by ICCS, a value we do not consider to be clinically significant. This is reinforced when, in the comparison between 2dVD and 3dVD values only a 12.6 mL difference was observed. Comparing 3dVD to 1dVD the difference found was also small (32.1 mL) and has a good correlation (r=0.82). In the authors opinion, this difference of a small volume (32.1 mL) in not clinically significant to the point of interfering in the diagnosis or treatment of these children and, to reinforce this statement, AVV was similar in all three groups (3dVD: 103.5 ± 39.2, 2dVD: 104.5 ± 46.2, and 1dVD 102.9 ± 46.9).

Different studies on the amount of days VD should be collected have suggested that 3 days are enough and trustable (14, 15). Although Homma et al. (16) ensure that longer periods can be more representative and with less data variation, Brown et al. (14) state that the length of the test period impairs patient compliance. Therefore, shorter periods of data collection on VD would be less burdensome and has less impact on the patient, tending to be more precise and reliable. Groutz et al. found that compliance decreased about 76% when VD was increased by 72 hours, while in a 24-hour diary, compliance was 92% (17).

Even with the latest revision and guidance from the ICCS (1), which reassures that performing the VD for 2 non-consecutive days is satisfactory, there are few studies in the literature that analyze the best period to evaluate the child's voiding pattern using the VD. Lopes et al. (11) assessed that the 2dVD is statistically and clinically comparable to a 3dVD and concluded that 2 days is a sufficient period to assess bladder capacity and liquid intake in children.

In our clinical experience, even after carefully explaining the importance and how to collect VD data by an experienced team of urotherapists, we have witnessed numerous complaints regarding the obstacles that hinder the dynamics of performing and filling it out. Lack of time, availability of an adult to assist with fulfilling it are the most reported complains. In addition, completing it for 3 consecutive days takes at least one school day for the child and a workday for the adult, even if including the weekend.

Mazurick and Landis (18) comparing 3 consecutive days of VD to one single day in women with interstitial cystitis, demonstrate that the measurements of VD during the week were not significantly different from those on weekends and also ensure that the VD can be reduced to a single day. Therefore, even with the differences in MVV volume found in the present study, we believe that only 1-day VD is sufficient for an adequate diagnostic evaluation and treatment guidance, especially for those low compliant families.

Because VD is a new event in the child's routine, as well as the child being enchanted by the idea of “peeing in the cup” and having more attention from parents, clinical impressions is that these changes could fantasize a higher frequency of voiding on the first day of VD, which was not true, being the frequency average similar between 1dVD, 2dVD, and 3dVD. The same was found by Lopes et al. (11), which showed that even with a higher average number in 2dVD, the voiding frequency agreed in 83.4% of cases with the 3dVD. Kwak et al. (19) also evaluated the 3-day diaries and questionnaire data of children with mono and non-monosymptomatic nocturnal enuresis and did not notice statistical differences in relation to the number of voiding.

Bladder capacity is an important parameter for assessing the lower urinary tract. Whether in the adult or pediatric population, studies have shown that VD has advantages over other methods, as it reveals the voiding routine more reliably (20, 21).

Researchers have suggested that bladder capacity is described as a factor involved in the etiology of PMNE, since children with enuresis have a relative decrease in EBC when compared to non-enuresis children (22, 23). As for the analysis of the relationship between MVV and EBC the present data demonstrate that 82.7% of the evaluated children have a MVV lower than the EBC. In our sample we have observed that MVV obtained by the VD was 67% of EBC in children presenting PMNE and 69% in those with OAB.

In opposite to the finds showed herein, Uluocak et al. (21), when comparing uroflowmetry with maximum cystometric capacity and MVV regarding the EBC in 84 children with OAB, realized that there is no significant difference between them and states that the VD was a reliable and non-invasive method for this group of patients.

Martinez-Garcia et al. (24) reviewing children's normal bladder capacity and measuring children's maximum normal voiding volumes and still comparing them with the usual formulas, concluded that the most accurate reference model for obtaining the MVV was based on records of VD and that even the simple linear formulas of Koff or Rittig are imprecise to be used in clinical situations. Following these findings, the present study also demonstrates that the formula suggested by ICCS to be used (1) is not precise in estimating bladder capacity in children with OAB and PMNE and both disorders have similar association between MVV and EBC, being MVV about 68% of EBC.

According to our findings, both 1dVD and 2dVD have a good sensitivity in evaluating MVV in children with PMNE and OAB, missing only a few individuals with very low bladder capacity (< 65% of EBC). In the other hand, doing only one-day VD has a lower specificity, which means that it includes a greater number of false positive individuals (those with lower capacity in 1dVD and normal capacity when evaluated with a 3dVD).

Although several measures were taken in order to avoid problems in data collection in the present study, such as team training on daily orientation, strict inclusion criteria, and patient evaluation, some weaknesses must be highlighted, such as the sample size and the non-standardization of measuring cups, as well as diaper weighting scales. In addition, the loss of incomplete diaries after the first meeting was not evaluated, since one of the inclusion requirements was the correct completion of the VD.

## CONCLUSIONS

Although MVV was lower by a small volume in 1dVD, we believe that 1-day VD is adequate to assess PMNE and OAB children, especially for those low compliant families. It shows high sensitivity and can provide a good snapshot of children's voiding habits. Bladder capacity in this population, evaluated by maximum voided volume is 67% and 69% of that obtained by the EBC formula proposed by ICCS for enuresis and OAB children, respectively.
